# P.R.L. Platelet Rich Lipotransfert: Our Experience and Current State of Art in the Combined Use of Fat and PRP

**DOI:** 10.1155/2013/434191

**Published:** 2013-09-28

**Authors:** V. Cervelli, I. Bocchini, C. Di Pasquali, B. De Angelis, G. Cervelli, C. B. Curcio, A. Orlandi, M. G. Scioli, E. Tati, P. Delogu, Pietro Gentile

**Affiliations:** ^1^Plastic Surgery Department, Tor Vergata University of San Salvatore, Lauro Place 15, 00186 Rome, Italy; ^2^Anatomic Pathology, Tor Vergata University of Rome, Rome, Italy

## Abstract

The authors report their experience about the use of P.R.L. PLATELET RICH LIPOTRANSFERT method (platelet rich plasma mixed fat grafting) in 223 patients affected by soft tissue defects (ulcers, Romberg syndrome, Hemifacial atrophy, loss of substance, and signs of aging). This paper introduces the reader to PRP therapy and reviews the current literature on this emerging treatment modality, showing at the current clinical use of PRP in plastic and reconstructive surgery, with description of innovative methods and future prospects. This technique provides a promising alternative to surgery by promoting safe and natural healing. Here recent studies concerning the use of PRP in the treatment of chronic ulcers and soft tissue defect are reviewed.

## 1. Introduction

In Europe, and more recently in the United States, an increased trend emerged in the use of autologous blood products to facilitate tissue regeneration and healing [1]. Platelet rich plasma is an autologous blood product by a selective removal or exchange of either packed red blood cells, leucocyte-rich or platelet-rich layers, or plasma, with a continuous flow blood separator machine. 

Its use of PRP has been firstly described in 1975 by Oon and Hobbs [2]. After its description, the first clinical application was performed by Ferrari et al. in 1987 [3], in open heart surgery, to avoid an excessive transfusion of homologous blood products. After that, its application has been safely used and documented in many fields, including maxillofacial surgery [4, 5], aesthetic plastic surgery [6–8], treatment of soft-tissue ulcers [9, 10], and regenerative surgery. 

In the 1999 Anitua [11] reported the preliminary clinical evidence of the beneficial effect of the use of P.R.P. in bone regeneration using plasmapheresis. After that he published a numerous reports about the use of PRP in tendon healing [12], orthopedic sport injuries [13], dental implants [14], and chronic cutaneous ulcers [15]. In 2009 he reported a study that described the fibroblastic response to the application of different preparations rich in growth factors [16].

This paper is a focused review of the literature about the clinical approaches of the use of PRP, describing also the experience of authors who coined the term of P.R.L. PLATELET RICH LIPOTRANSFERT (method of mix and use of platelet rich plasma with fat grafting) and methods of application updated.

*Scope of the Paper.* The aim of this paper is to analyze the efficacy of PRP mixed to fat tissue in wounds and soft tissue defects. The authors evaluate here the clinical efficacy of different concentration of PRP and compare also the results obtained with a control group treated not with PRP or some other blood derivate. This paper would also provide a concise compilation of recent advances in this field.

## 2. Materials and Methods

The authors used [17] several concentration of PRP, from 0.2 mL to 0.5 mL, to be added to fat tissue for wounds and soft tissue defects. The objective of the study was to identify the optimal concentration (quantity in mL in clinical practice) of platelet gel that might be added to each mL of adipose tissue harvested by the method of Coleman [18, 19]. The authors V. Cervelli and P. Gentile coined a term of P.R.L. PLATELET RICH LIPOTRANSFERT to describe a method of mix and use of platelet rich plasma with fat grafting.

### 2.1. Patients

From July 2009 to July 2012, 223 patients (134 males and 89 females), aged from 18 to 75 years (mean  age = 36.6  years), underwent to infusion of P.R.L. PLATELET RICH LIPOTRANSFERT in the Department Plastic and Reconstructive Surgery of the University of Tor Vergata, Rome. The patients were divided into three groups:

 Group A: composed of 132 patients affected by soft-tissue defects with loss of volume and elasticity, associated with signs of aging (70 males and 62 females);
 Group B: composed of 87 (63 males and 24 females) patients affected by chronic venous lower extremity ulcers;
 Group C: composed of 4 patients affected by Romberg syndrome and Hemifacial atrophy (1 male and 3 females).

Patients of Group B (venous low extremity ulcers) are affected by the following comorbidity: dislipidemie in 16 patients (18%), cardiological disease in 15 patients (17%), hypertension in 10 (12%), diabetes in 32 (37%), arteriopathy of lower extremity in 4 (5%), and no comorbidity in 10 patients (12%).

**Figure 1:**
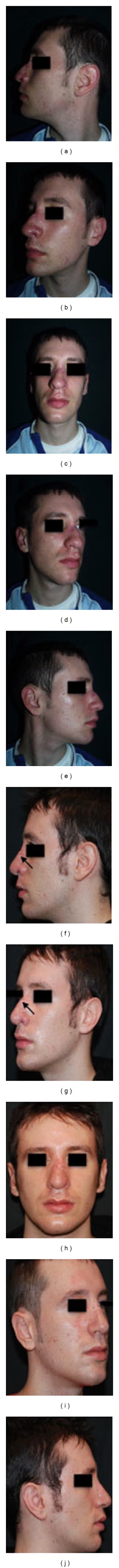
Analysis of patients affected by outcomes of scars. (a) Preoperative situation in lateral left projection. (b) Preoperative situation in 3/4 left projection. (c) Preoperative situation in frontal projection. (d) Preoperative situation in 3/4 right projection. (e) Preoperative situation in lateral right projection. (f) Postoperative situation in lateral left projection. (g) Postoperative situation in 3/4 left projection. (h) Postoperative situation in frontal projection. (i) Postoperative situation in 3/4 right projection. (j) Postoperative situation in lateral right projection.

The authors compared their results with three homogeneous control groups, presenting these features:

 Control Group A: 132 patients affected by outcomes of scars (70 males and 62 females) treated with fat injection only (Figure 1);
 Control Group B: 87 patients affected by venous low extremity ulcers (60 males and 27 females) treated with curettage and application of biomaterials (hyaluronic acid and collagens);
 Control Group C: 4 patients affected by Romberg Syndrome (2 males and 2 females) treated with fat injection only.

General exclusion criteria were: platelet disorders, thrombocytopenia, antiaggregating therapy, bone marrow aplasia, uncompensated diabetes, sepsis, and cancer. Local exclusion criteria were infection or diastasis. 

### 2.2. Clinical Evaluation Methods

Tissue regeneration was evaluated by the analytical comparison of pre- and postoperative images. In addition three methods for the evaluation of outcomes were used: (1) team evaluation, (2) NMR (nuclear magnetic resonance) and Ultrasound, (3) patient self-evaluation. 

The Team evaluation is an evaluation method based on clinical observation, using a scale of six values (excellent, good, discreet enough, poor, and inadequate). The factors/variables, considered were pigmentation, vascularization, pliability, thickness, itching, and pain.

The patient evaluation is an evaluation method based on clinical observation, using a scale of six values (excellent, good, discreet enough, poor, and inadequate). 

In addition, in complicated cases, a high-resolution CT scan with 3D imaging for a better view of the anatomical structures was performed. In the venous low extremity of patients affected by ulcers a biopsy punch 2–4 mm of diameter was collected.

A followup of the patients was performed at the second and fifth weeks and at 3, 6, and 12 months and then annually. 

### 2.3. Platelet Rich Plasma Preparation

The traditional PRP preparation consisted in a slow centrifugation; platelets remain suspended in the plasma, while the leukocytes and erythrocytes are displaced to the bottom of the tube. A rapid centrifugation can cause mechanical forces and can raise the temperature, inducing changes in the ultrastructure of platelets that stimulate partial activation, with a consequent loss of its content [20].

Generally, the authors prepared PRP (using centrifuge at 1100 g for 10 min) from a small volume of blood (18 cc) according to the Cascade [17, 20, 21] method and in all cases with approval of Transfusional Service.

Anitua et al. [11, 14–16, 22] reported the use of two centrifuges; sample of blood was collected into 3.8% (wt/vol) sodium citrate and was centrifuged either at 4500 g for 12 min at 4°C to obtain PP-plasma or at 460 g for 8 min to obtain PR-plasma. Calcium chloride was added to PP- and PR-plasma of each donor at a final concentration of 22.8 mM [23]. 

Standard cell separators and salvage devices can be used to produce platelet-rich plasma [24]. These devices operate on a unit of blood and typically use continuous-flow centrifuge bowl or continuous-flow disk separation technology and both a hard (fast) and a soft (slow) spin, yielding platelet concentrations from two to four times baseline [25, 26]. Such devices include the CATS (Fresenius, Wilmington, DelDE), sequestra (Medtronic, Minneapolis, MN) and Haemonetics Cell Saver 5 (Haemonetics Corp., Braintree, MA), [25–27].

Many surgical procedures require the use of relatively small volumes of platelet-rich plasma [28]. 

Consequently, small, compact office systems that produce approximately 6 mL of platelet-rich plasma from 45 to 60 mL of blood have been developed [29–31].

There are many of such systems, including the GPS (Biomet, Warsaw, IN), the PCCS (Implant Innovations, Inc., Palm Beach Gardens, FL), the Symphony II (DePuy, Warsaw, IN), the SmartPReP (Harvest Technologies Corp., Norwell, MA), and the Magellan (Medtronic, Minneapolis, MN) [25, 27, 29–31]. Although all operate on a small volume of drawn blood (45 to 60 mL) and on the principle of centrifugation, these systems differ widely in their ability to collect and concentrate platelets, with approximately 30 to 85 percent of the available platelets collected and from a less than 2-fold to an approximately 8-fold increase in the platelet concentration over baseline [25, 26].

The authors used in the personal experience Cascade-Fibrinet (Cascade Medical Enterprises, Plymouth, Devonshire, UK), Vivostat (Vivostat A/S, Borupvang 2, DK-3450 alleroed, Denmark), and Regen (Regen Lab, En Budron B2, CH-1052 Le Mont-sur-Lausanne, Switzerland).

In general, a lot of systems do not concentrate on the plasma proteins of the coagulation cascade [25, 27]. The concentration of plasma proteins above baseline can be achieved through secondary ultrafiltration, as done with the UltraConcentrator (Interpore Cross, Irvine, CA) and the Access System (Interpore Cross), in which the buffy coat collected from a centrifugation stage is passed through hollow fibers with an effective pore size of 30 kDa. 

With this system, up to two-thirds of the aqueous phase is removed by filtration; thus, the concentrations of the retained plasma proteins and formed elements are correspondingly increased [32–34]. 

### 2.4. Platelet Rich Lipotransfer Preparation

Fat harvesting was performed in the same moment of the PRP preparation. We harvested fat tissue in the abdominal region using some specific cannula, with diameters of 2 to 3 mm and 1.5 mm, for grafting (Coleman Kit, Tucson, AZ) [15, 16, 18, 20, 34]. We took the plungers of syringes and closed them with their caps then positioned them flatly in the sterile centrifuge, to maintain asepsis. The syringes were processed for 3 min at 3,000 rpm/min [17, 18, 20, 34]. This purified body fat combined with PRP was put in 1 mL syringes and aseptically reinjected using the specific microcannulas (Coleman Kit, Tucson, AZ) [18, 34] to implant it into the area to be treated. Skin incisions about 2 mm in diameter to permit passage of the cannula were made using a number 11 scalpel blade. The implant location destined to receive the implant was selected by an accurate study of the necessary corrections [17, 21, 24, 35]. Fat tissue combined with PRP was implanted at different levels in small tunnels around the margins created earlier by forcing the cannula with precise controlled movements. A small quantity of fat cells was laid, one or two at a time, during the exiting movement of the cannula to create a large grid to correct the vascular development around each fat cell. Layers of the aligned single cells were laid to increase the contact surface between the receiving tissue and the implant. This technique was of fundamental importance in allowing each single layer deposited to survive through the few days necessary for the growth of the blood vessels that would nourish them permanently [9, 36]. We closed the access incisions with 5–0 nylon stitches, and a compressive bandage was not applied.

The fat volume was injected in the selected areas according to the defect to be corrected; in the scars the volume ranges between 10 cc and 80 cc, and in the ulcers ranges between 5 cc and 50 cc, in the Romberg syndrome ranges between 60 cc and 140 cc.

## 3. Results

### 3.1. *In Vivo*: Influence of Platelet-Rich Plasma in Tissue Regeneration and during Fat Grafting Surgical Procedures

Previously the authors published in Tissue Eng 2009 [20] their experience about the application of platelet rich plasma in plastic surgery. The authors added 0.5 mL or 0.4 mL of PRP per each mL of fat tissue (Figure 4). They observed that 61.1% and 88.9% of chronic lower extremity ulcers 100% reepithelization during an 7.1 and 9.7-week (average) course of twice-daily wound treatment with PRP suspended on a collagen base, respectively, compared with 40 and 60% of the first control group (*n* = 10), respectively, treated with hyaluronic acid and collagen medication. 

When the authors added 0.3 mL of PRP per each mL of fat tissue, they observed 55.5% and 72.2% of chronic lower extremity ulcers 100% reepithelization during an 7.1 and 9.7-week average.

When the authors added 0.2 mL of PRP per each mL of fat tissue, they observed 44.4% and 66.6% of chronic lower extremity ulcers 100% reepithelization during an 7.1 and 9.7-week average. We also observed in patients affected by soft tissue defect treated with reconstructing three dimensional projection by fat grafting and PRP at concentration of 0.5 mL or 0.4 mL per each ml of fat tissue, a 70% maintenance of contour restoring and three-dimensional volume after 1 year, and only 31% in control patients (*n* = 10) treated 200 with only fat grafting; when we used 0.3 mL and 0.2 mL of PRP per each mL of fat tissue, we observed a 62% and 50% maintenance of contour restoring after 1 year.

### 3.2. *In Vitro*: Adipose Tissue Derived Stem Cells Isolation and Expansion

Liposuction aspirates were washed three times with phosphate-buffered saline (PBS), suspended in an equal volume of PBS and 0.1% collagenase type I (C130; Sigma-Aldrich, Milan, Italy), and prewarmed to 37°C [37, 38]. Adipose tissue was placed in a shaking water bath at 37°C with continuous agitation for 60 min and centrifuged for 10 min at 600 g at room temperature. The supernatant, containing mature adipocytes was aspirated. The stromal vascular fraction pellet was resuspended in erythrocyte lysis buffer (155 mM NH_4_Cl, 10 mM KHCO_3_, and 0.1 mM EDTA) and incubated for 5 min at room temperature [28]. After centrifugation for 5 min, the pellet was resuspended in few *μ*L of growth medium and passed through a 100 *μ*m Falcon strainer (Becton & Dickinson, 129 Sunnyvale, CA, USA), and cellular population was counted using haemocytometer. In twelve patients randomly selected, nucleated SVF cells were 317,578 + 25,645 per mL of lipoaspirate. Then digestion was plated in DMEM (Euroclone, Pavia, Italy) supplemented with 10% (v/v) fetal bovine serum (FBS; Euroclone, Pavia, Italy), 2 mM L-glutamine, 100 U/mL penicillin, 100 g/mL streptomycin and 0.25 g/mL fungizone (Invitrogen, Milan, Italy), at a density of 2500–5000 cell/cm^2^ of surface area. This initial passage of the primary cell culture was referred as passage 0 (P0). After 48 hours of incubation at 37°C at 5% CO_2_, the cultures were washed with PBS and maintained in stromal media until they achieved 75%–90% confluence [37, 38]. Adipose tissue derived stem cells (ASCs) were passaged by trypsin (0.05%) digestion and plated at a density of 5,000 cells/cm^2^ (P1). Medium was changed every 3 days. To demonstrate cultured ASCs differentiation potential, adipogenic and osteogenic differentiation was obtained in 3rd passage confluent cells according to previously published methods [39]. To assess mineralization corresponding to osteogenic differentiation, intracellular calcium deposits were stained with von Kossa. Images of cultured cells after Oil Red O and von Kossa staining were obtained through a digital telecamera (Nikon, model 152 Dxm1200F), connected to a computer utilizing Nikon ACT-1 software with a light microscope at 200x magnification.

### 3.3. Platelet-Rich Plasma Concentrations Increases Adipose Tissue Stem Cells Number *In Vitro*

ASCs at the third passage were seeded at a density of 5000 cells/cm^2^ in 24-well plates and incubated for 24 h in DMEM containing 10% FBS. Medium was then replaced with DMEM containing 0.1% FBS for starvation. After 24 h the medium was changed, for the treatment, with DMEM 10% FB (control) and DMEM 10% FBS 5% PRP. The medium containing the respective supplements was replaced every 2 days. After 0, 2, 4, 6, and 8 days of culture, cells were digested with 0.25% trypsin solution and then counted, with trypan-blue exclusion, using hemocytometer. Cell viability by trypan blue exclusion was consistently more than 98%. The reported results were the mean value of triplicate samples. Each assay was performed twice. As reported previously [20], PRP induced an increase of ASC number without any morphological changes compared with control. There was a statistically significant increase, by around fourfold, at 4 and 6 days, when cells were preconfluent (*P* < 0.02). After 8 days, at confluency, there was a threefold increase of adipose ASC number in PRP cultures compared to controls. Oil Red O staining did not reveal any significant difference in intracytoplasmic lipid accumulation compared with PRP-treated and control ASCs. The authors reported the effects of platelet-rich plasma on proliferation of human adipose-derived stem cells (Figure 5). In Table were presented growth curves that show the dose-dependent increase of proliferation with PRP. In detail growth curves documented that PRP increased ASC proliferation in a dose-dependent manner (EC50 = 15.3 ± 1.3% vol/vol), with a fourfold increase of cell number at 5% (vol/vol) dosage compared with control after 4 days (*P* < 0.05). 

## 4. Discussion and PRP Therapies

The present findings demonstrated that the different concentration of PRP (ranging from 0.2 mL to 0.4 mL) influence the *in vitro* adipose derived mesenchymal stem cell proliferation. Higher concentration was observed at a concentration of 0.4 mL. When 0.4 or 0.5 mL PRP was injected mixed with 1 mL of centrifuged fat, it favoured growth and restored fat volume maintenance, confirming data observed with other surgical procedures, including periodontal [40, 41] and oral surgery [4, 22, 42, 43], maxillofacial surgery [4, 5], aesthetic plastic surgery [6–8], spinal fusion [44–46], heart bypass surgery [47], and treatment of soft tissue ulcers [9, 10] (Figure 3).

PRP, being produced during surgical procedures under sterile conditions, is easy to produce and safe to use; moreover, PRP is lacking of surface antigens, responsible of potential allergic reactions [48]. 

Our results clearly documented that the use of PRP during fat grafting favours adipose tissue maintenance and survival. Moreover, our *in vitro* data are in accordance with the hypothesis that PRP stimulates adipose tissue regeneration, as demonstrated in controlled animal studies for both soft and hard tissues [18]. In addition, in comparing to lipofilling [19, 34, 49] where fat cells are laid in rows without solution of continuity, implant survival is likely derive from reduction of fat necrosis due to improved neoangiogenesis in the implanted area.

The authors feel that there are new issues in the literature about the selection of the most appropriate regenerative methods. Indeed, there are many publications regarding the use of PRP with/without fat graft in plastic and reconstructive surgery; the authors divided the review of the application of PRP in the following pathologies.

### 4.1. PRP in the Treatment of Chronic Ulcers

About this topic there is not paper in the literature describing the use of PRP mixed with fat graft but a large number of paper based on use of only PRP or PRP with biomaterials (hyaluronic acid and collagen). Nonhealing cutaneous wounds represent a challenging problem and are commonly related to peripheral vascular disease, infection, trauma, neurologic, and immunologic conditions, as well as neoplastic and metabolic disorders.

These chronic ulcerative wounds represent significant impact both psychologically and socioeconomically. An analysis of the surfaces of chronic pressure wounds (decubitus ulcers) revealed a decreased growth factor concentration compared with acute wound [36]. In a study by Crovetti et al. [50], 24 patients with chronic skin ulcers were treated with a series of PRP Gel treatments. Nine patients demonstrated complete wound healing. In these subgroups, one wound reopened at 4 months. There were two reports of wound infection, both with positive *Staphylococcus Aureus* which were successfully treated with oral antibiotics. There were no adverse effects encountered and all patients noted decreased pain. 54 242 Another wound study by McAlee et al. [51], involved 24 patients with 33 chronic nonhealing lower extremity wounds.

Patients failed conservative treatment for 6 months with a lack of reduction of surface area. The wounds were injected with PRP every 2 weeks. Successful wound closure and epithelization was obtained in 20 wounds. The mean time for closure was 11.15 weeks. Five wounds displayed no improvement [51]. These findings were particularly significant because all patients had failed previously available treatment methods.

Rigotti et al. [52], reported the use of only PRP in skin and soft-tissue losses on the basis of its bactericidal and cell proliferation promoting properties.

**Figure 2:**
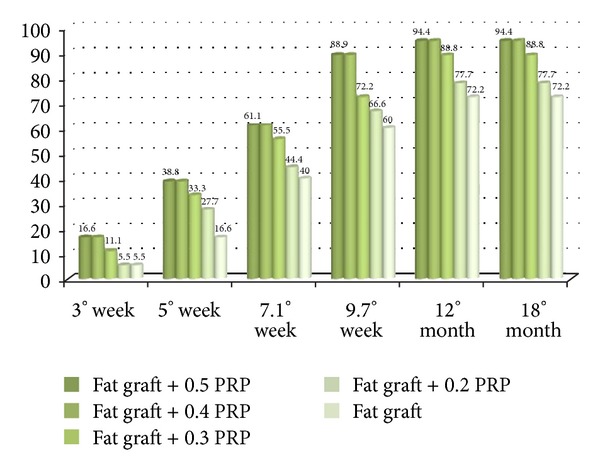
Bar graphs showing effects of PRP at different concentrations on percentages of skin chronic ulcer reepithelization.

**Figure 3:**
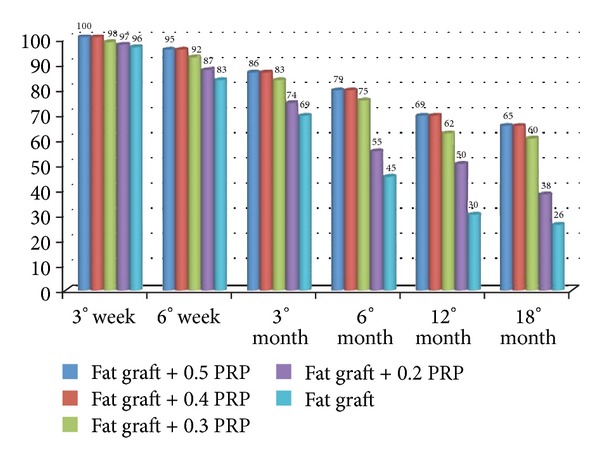
Bar graphs showing effects of PRP at different concentrations on percentages of maintenance of restored fat.

**Figure 4:**
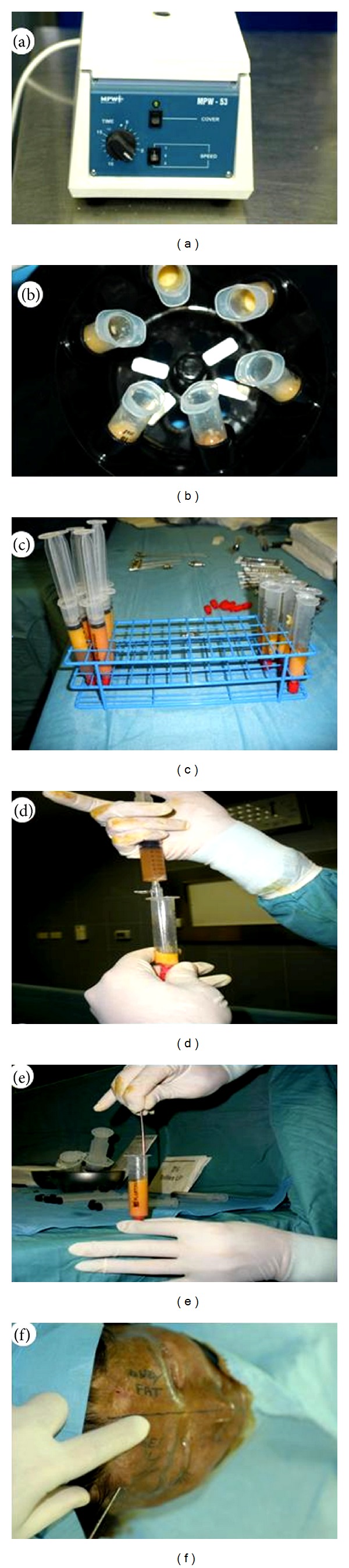
P.R.L. PLATELET RICH LIPOTRANSFERT procedure. (a) Platelet rich plasma preparation according to Cascade centrifuge; (b) fat graft preparation according to Coleman Centrifuge; (c) purified fat graft after centrifugation; (d) addition of PRP to purified fat graft; (e) mix of 0.4 mL of PRP with 1 mL of fat graft in a 10 mL luer-look syringes (P.R.L. PLATELET RICH LIPOTRANSFERT); (f) injection of P.R.L. PLATELET RICH LIPOTRANSFERT according to lipostructure technique.

**Figure 5:**
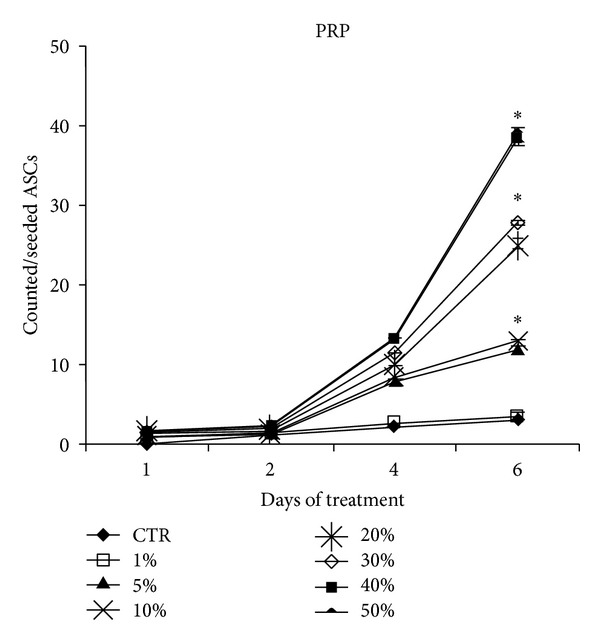
Effects of platelet-rich plasma on proliferation of human adipose-derived stem cells. Growth curves show the dose-dependent increase of proliferation with PRP.

The authors described the use of fat grafting combined with platelet-rich plasma for chronic lower-extremity ulcers [21], evaluating the healing rate of skin chronic ulcer, according method of Kazakos [49], with minor modifications (Figure 2).

### 4.2. Use of PRP in Soft Tissue Defect

Powell et al. [53], describe anti-inflammatory properties with reduced edema and ecchymosis associated with the autologous platelet gel in eight women after deep-plane rhytidectomy (face lifting).

PRP was also shown to be effective in stopping capillary bleeding in the surgical flaps of a series of 20 patients undergoing various cosmetic surgery (face lift, breast size changes, or neck lifts) reported by Man [54].

Anderson [55] reported the use of fat tissue without PRP in patients affected by Romberg syndrome disease and facial defects. In addition, they recently reported interesting cases treated with rhytidoplasty combined with pursing plication suspension sutures and lipoinjection [56].

Recently, the authors described the use of fat graft with platelet rich plasma [35] and with only lipostructure technique [36] in patients affected by Romberg syndrome.

Lipostructure evolved from lipofilling and is better known as Coleman's technique [18, 19]. In fact Coleman is the pioneer in the use of fat graft with lipostructure in the facial recontouring [19].

In addition Yoshimura et al. [57] describe a new methods and new technologies in the use of fat grafting. In fact, they use a cell-assisted lipotransfer (CAL) for cosmetic breast augmentation using adipose derived stem/stromal cells. In CAL, autologous adipose derived stem (stromal) cells (ASCs) are used in combination with lipoinjection. A stromal vascular fraction (SVF) containing ASCs is freshly isolated from half of the aspirated fat and recombined with the other half. This process converts relatively ASC poor aspirated fat to ASC-rich fat.

A new aim could be the use of SVF isolated from half of fat tissue mixed with platelet rich plasma and recombined with the other half.

### 4.3. PRP in the Treatment of the Scars

Azzena et al. [58] hypothesized that autologous platelet-rich plasma (APRP) could be used as an *in vivo* adipocyte delivery system to favour cell survival and to stimulate early recruitment of microcapillaries to the site of implantation and treated a patient affected by adherent scar.

Zocchi [59] identified a few points for the readers to consider. (1) The cutting edge for fat transplantation is to treat the fat tissue not only as a volume replacement but also as a tissue regenerator, as a vehicle carrying its stromal fraction very rich in precursor cells, and as stem cells into the injection site. (2) It is mandatory to remember that this type of tissue manipulation should be performed only by well trained surgeons in well equipped and reliable facilities. (3) The use of platelets gel even for other clinical applications is still controversial, still not so easy for daily practice, still limited by laws and regulations, and still quite expensive.

## 5. Current and Future Challenges

This work suggest two fundamental points: first, PRP added in concentration of 0.4 mL (40%) per each mL of fat tissue favours an optimal ASCs proliferation with correct architectural adipocytes distribution, 58 298 better cell-to-cell interaction, adipose tissue growth, and differentiation from ASCs; this offers early protection from surrounding inflammatory events [60]. Secondly, PRP-induced early development of neoangiogenetic microcapillary network [61] favours the delivering of proper nutrient and oxygen levels to grafted cells. Actually, there are the several questions left to be solved in the immediately future of this exciting therapy: first, we shall have to explore which of the growth factors that PRP contain is best for tissue regeneration in plastic and reconstructive surgery; second, standardization of the use of PRP mixed with fat graft as a therapeutic product, and third, clinical and *in vitro* evaluation of the products as a insulin in/out addition of PRP during the use of fat graft.

Authors suggest that growth factors present in the PRP play a role in improving tissue healing. VEGF should be used to implement the neoangiogenesis in patients affected by vascular disease that has generated a loss of substance and which prevents or delays the healing process; the FGF may be used to implement the rejuvenation of the tissues and the PDGF-BB to implement chemotaxis and mitogenic effects. The perspective future start from the paper of Katz et al. [62] that describes the effect of human stromal cells derived from adipose tissue (ADSC) on pancreatic tumor cell proliferation.
